# Early rescue oocyte activation at 5 h post-ICSI is a useful strategy for avoiding unexpected fertilization failure and low fertilization in ICSI cycles

**DOI:** 10.3389/fendo.2023.1301505

**Published:** 2024-01-04

**Authors:** Lintao Xue, Shikai Wang, Pingpin Wei, Haifang Liu, Xianbao Mao, Jie Qin, Yaoxuan Li, Xiaohui Zhang, Zhengda Li, Yueyue Huang, Liangshi Chen, Wen Shi, Liling Liu

**Affiliations:** Reproductive Medical and Genetic Center, The People’s Hospital of GuangXi Zhuang Autonomous Region, Nanning, China

**Keywords:** intracytoplasmic sperm injection, fertilization failure, artificial oocyte activation, second polar body, time lapse

## Abstract

**Introduction:**

Attempts to artificially activate unfertilized oocytes at 24 h post intracytoplasmic sperm injection (ICSI) have generally resulted in poor outcomes. This study aims to explore a new strategy for early judgement and rescue activation of unfertilized oocytes at 5 h post ICSI to avoid unexpected fertilization failure (UFF) or unexpected low fertilization (ULF) in ICSI cycles.

**Methods:**

Firstly, time-lapse data from 278 ICSI cycles were retrospectively analyzed to establish an indicator for fertilization failure prediction. Secondly, 14 UFF and 20 ULF cycles were enrolled for an observational study, early rescue oocyte activation (EROA) was performed on oocytes without post-ICSI Pb2 extrusion to investigate fertilization efficiency, embryo development and clinical outcomes.

**Results:**

The average time to Pb2 extrusion post-ICSI was 3.03±1.21 h, 95.54% of oocytes had extruded Pb2 before 5 h, and the sensitivity and specificity for monitoring Pb2 extrusion at 5 h by time-lapse imaging to predict fertilization were 99.59% and 99.78%, respectively. Early rescue activation of oocytes with no Pb2 extrusion resulted in acceptable fertilization and embryo developmental outcomes, in terms of the fertilization rate (75.00, 72.99%), 2PN fertilization rate (61.36, 56.93%), good-quality embryo rate (42.59, 50.00%), blastocyst formation rate (48.28, 46.03%), good-quality blastocyst rate (34.48, 33.33%), and oocyte utilization rate (36.36, 27.74%), for both UFF and ULF cycles. The clinical pregnancy, embryo implantation, and early miscarriage rates in the rescue oocyte activation group did not significantly differ from those in the Pb2 extrusion group. Fourteen unexpected fertilization failures and 20 low fertilization ICSI cycles were rescued and resulted in clinical pregnancy rates of 40.00% (4/10) and 57.14% (8/14), respectively.

**Conclusions:**

This study demonstrates that monitoring Pb2 extrusion by time-lapse imaging can accurately predict fertilization outcomes, suggesting that early rescue oocyte activation at 5 h post ICSI is an effective strategy for avoiding unexpected fertilization failure and low fertilization in ICSI cycles.

## Introduction

Intracytoplasmic sperm injection (ICSI) is the most effective treatment for severe male infertility and previous fertilization failure, and the procedure involves the artificial injection of a single spermatozoon into the oocyte using micromanipulation techniques. Although the reported fertilization rate in ICSI cycles is higher than that in conventional *in vitro* fertilization (IVF), unexpected fertilization failure (UFF) or unexpected low fertilization (ULF) is still a risk event for ICSI treatments (1). It has been reported that total fertilization failure occurs in 1-5% of ICSI cycles (2), and the average fertilization rate is approximately 65-80% (3). Thus, spermatozoon injection into oocytes can overcome the sperm–oocyte binding barrier but cannot ensure complete fertilization when there are sperm defects or oocyte abnormalities present (4, 5). More importantly, there is currently no technique that can accurately predict the occurrence of fertilization failure in ICSI cycles. Patients in this situation can only accept and attempt the next cycle, which increases the medical burden and wastes precious oocytes obtained during a superovulation cycle.

Oocyte activation deficiency (OAD), such as a lack of Ca^2+^ oscillation, and inadequate oocyte activation, are the main contributors to fertilization failure (6). Multiple studies have shown that artificial oocyte activation (AOA) can successfully increase the plasmatic calcium concentration and activate the oocyte, but most studies on AOA treatment are performed in the cycle after the experience of fertilization failure (7). Few studies have attempted to perform AOA to rescue oocytes with fertilization failure in the current cycle. Two studies have indicated that performing AOA on one-day-old oocytes, even 68 h after ICSI, may enable normal fertilization of unfertilized oocytes, but the blastocyst formation outcomes are extremely poor (8, 9). The main factors leading to such poor developmental outcomes include cytogenetic abnormalities occurring with the increasing age of the oocytes. Shibahara et al. reported that artificial rescue oocyte activation at 2.5-6 h after ICSI leads to a blastocyst formation rate of 48.9% and implantation rates that are similar to those of conventional ICSI cycles (10). These results suggest that early rescue oocyte activation (EROA) may be a valuable strategy for rescuing unfertilized oocytes and improving the oocyte utilization rate.

However, the clinical efficiency of EROA, especially the clinical value of EROA in avoiding unexpected fertilization failure and low fertilization in ICSI cycles, needs to be further explored. In this study, we first investigated the relationship between post-ICSI extrusion of the second polar body (Pb2) via monitoring by time-lapse imaging and fertilization and evaluated embryo developmental outcomes to establish an indicator to predict fertilization failure. Second, we performed EROA on oocytes without post-ICSI Pb2 extrusion to investigate fertilization efficiency, embryo development and clinical outcomes.

## Materials and methods

### Patients and study design

**Figure 1:**
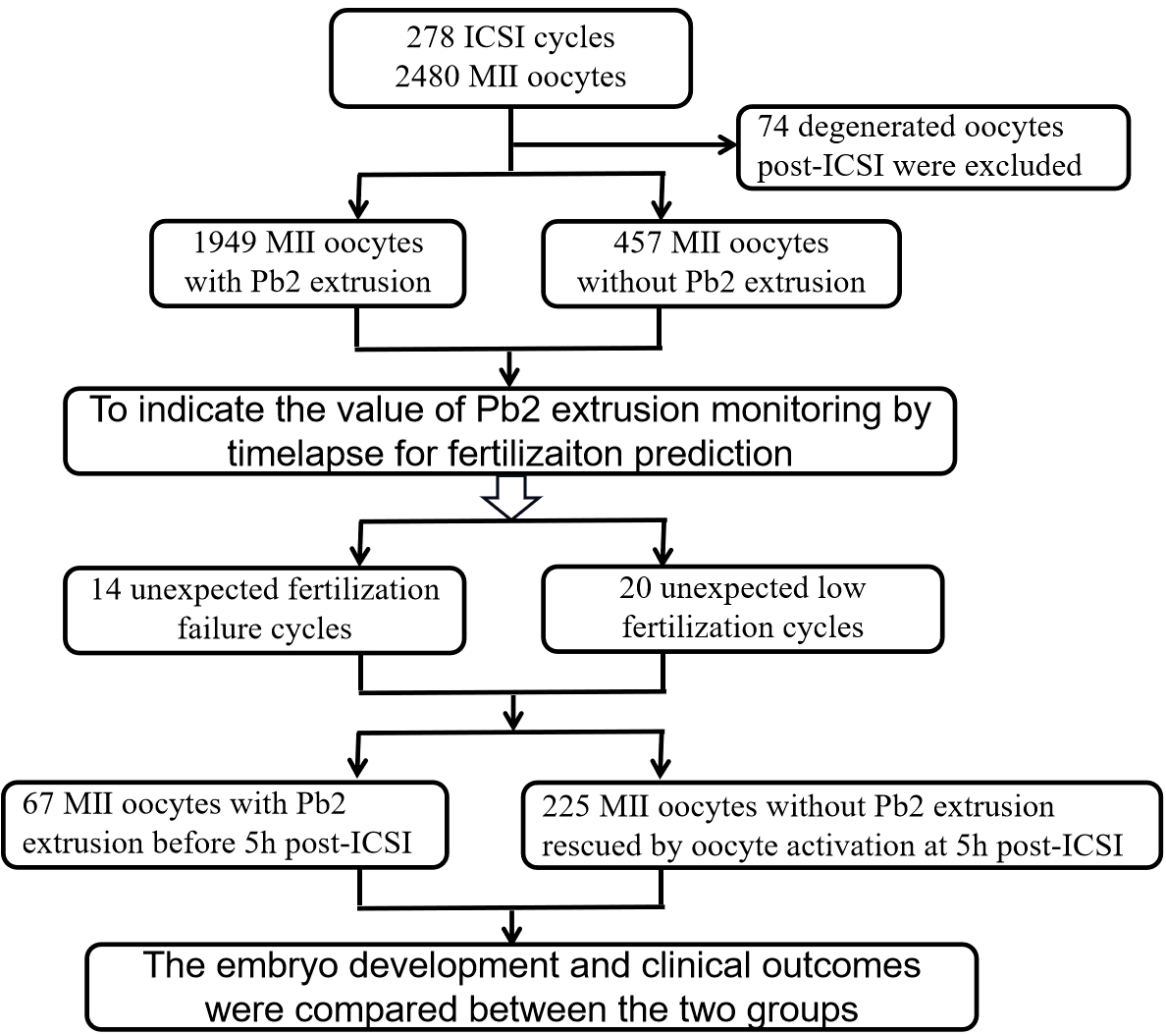
Flow chart of the study design.

This retrospective study consisted of two parts (**Figure 1**). First, we retrospectively analyzed the time-lapse data of patients who received ICSI treatment in the Reproductive Medicine and Genetic Center of the People’s Hospital of Guangxi Zhuang Autonomous Region from January 2017 to May 2019. The distribution of Pb2 extrusion time and its relationship with fertilization and embryonic developmental outcomes were analyzed to confirm the optimal time point for EROA. Then, an observational study was performed to collect UFF and ULF cycles from June 2019 to July 2023. The inclusion criteria of UFF and ULF cycles was patients who received ICSI treatment while all embryos cultured in time-lapse system.UFF cycles were defined as all ICSI oocytes without Pb2 extrusion at 5 h post-ICSI, while ULF cycles were defined as ≤50% of ICSI oocytes without Pb2 extrusion after referring to previous studies (1, 6). All oocytes without Pb2 extrusion underwent EROA at 5 h post-ICSI, and the other oocytes that spontaneously extruded Pb2 were used as the control group. Fertilization, embryonic development, and clinical outcomes were compared between the different groups. Patients who underwent AOA immediately after ICSI due to previous fertilization failure or other reasons were excluded. Each enrolled patient signed an informed consent form, and the study was approved by the Ethics Committee of the People’s Hospital of Guangxi Zhuang Autonomous Region on 26 February 2021 (Approval No: LL-KY-SY-2021-16).

### Oocyte retrieval and ICSI

Controlled ovarian hyperstimulation was performed using the GnRH agonist, GnRH antagonist, progestin-primed ovarian stimulation, mini-stimulation or other protocols. Oocytes were retrieved 34–36 h after the administration of HCG by transvaginal aspiration under ultrasound guidance. The cumulus cells surrounding the retrieved oocytes were removed mechanically with 80 IU/ml hyaluronidase after 3 h of preculture in G-IVF PLUS™ (10136, Vitrolife). All MII oocytes were confirmed using an inverted microscope, and then ICSI was performed. The injected MII oocytes were transferred into G-1 PLUS™ (10128, Vitrolife), covered with mineral oil in a Primo Vision dish (9-well or 16-well, Vitrolife) and cultured in a Primo Vision (Vitrolife, Budapest, Hungary) incubator at 37°C, 6% CO_2_, and 5% O_2_. Images at 5-min intervals were taken to monitor Pb2 extrusion, fertilization and embryo developmental processes.

### EROA procedure

**Figure 2:**
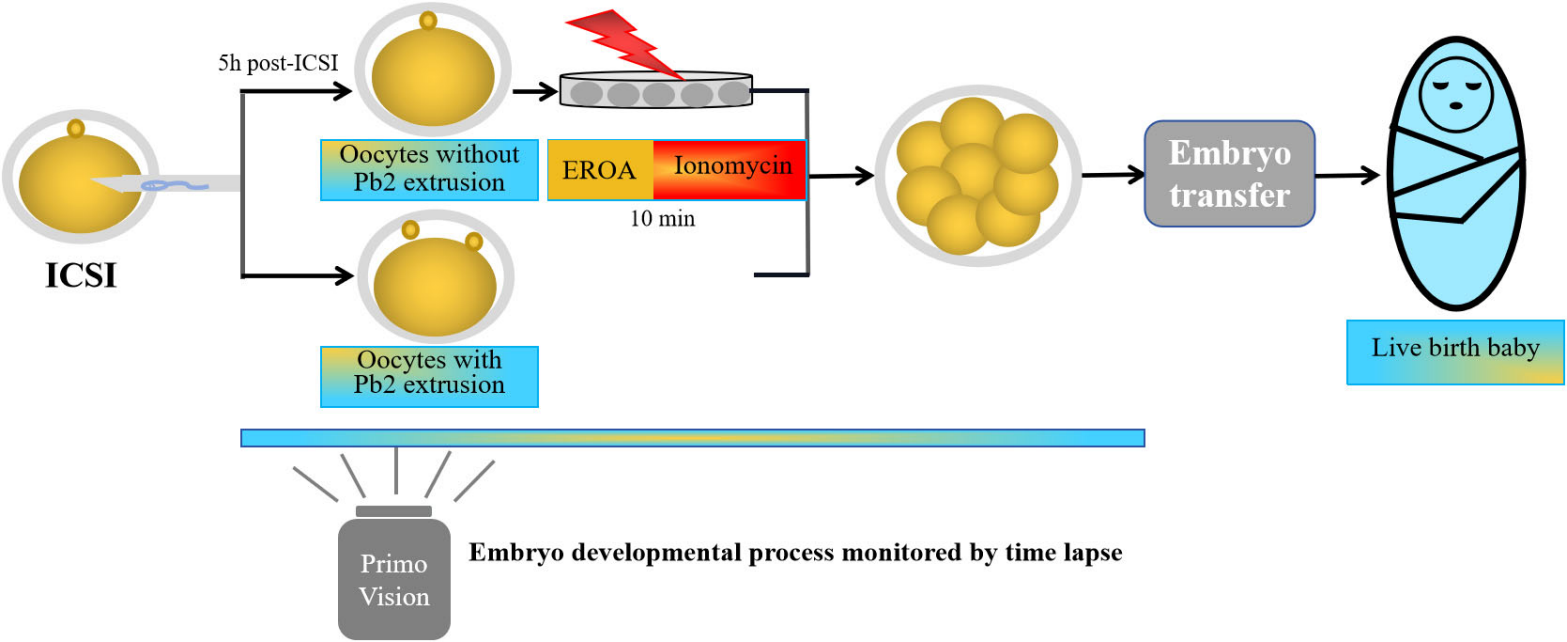
A scheme describing early rescue oocyte activation process is shown.

The dynamic process of Pb2 extrusion was monitored by time lapse, and spontaneous Pb2 extrusion was defined as the extrusion of the entire polar body from the cytoplasm post-ICSI. Oocytes without Pb2 extrusion before 5 h post ICSI by time lapse monitoring were defined as fertilization failure, and then EROA was performed. Oocytes with fertilization failure were transferred into 50 μl G-IVF PLUS™ (10136, Vitrolife) containing 10 μmol/L ionomycin. Oocytes were activated for 10 min and then thoroughly washed and transferred into Primo Vision (Vitrolife, Budapest, Hungary) at 37°C, 6% CO_2_, and 5% O_2_. The EROA procedure is described in **Figure 2**; **Supplementary Video 1**.

### Embryo culture and transfer

On day 1, fertilization status was evaluated by observing pronuclei in the cytoplasm, and the observation of two pronuclei indicated normal fertilization. On day 3, the quality of cleavage-stage embryos was evaluated based on factors such as cleavage speed, uniformity of blastomeres, and fragmentation rate. Embryos derived from normally fertilized oocytes with 7-9 cells and less than 10% fragmentation were defined as high-quality embryos. On day 5/6, the quality of blastocysts was evaluated using the Gardner scoring system, and blastocysts that were better than 3BC or 3CB were defined as high-quality blastocysts.

On day 3 or 5, embryos were transferred into the uterine cavity. After 14 days, the serum hCG concentration was tested, and serum hCG >10 mIU/ml was defined as chemical pregnancy. Clinical pregnancy was defined as the presence of an intrauterine gestational sac, and the heartbeat was confirmed by ultrasound examination at the 4th week after embryo transfer. Early miscarriage was defined as pregnancy loss before 12 weeks. Ongoing pregnancy was defined as an intact pregnancy without termination of pregnancy before the 12th week of gestation. Live birth was defined as any birth event in which at least one baby was born alive and survived for more than 1 month.

### Statistical analysis

Statistical analysis was carried out using SPSS 17.0 software (IBM, SPSS Statistics). The quantitative data were normally distributed as determined by the Kolmogorov–Smirnov test and are presented as the mean ± standard deviation, and unpaired Student’s *t* test or Mann–Whitney nonparametric tests were used to compare the mean values as appropriate. Categorical data are presented as percentages (%), and the chi-square test was used to compare the rates between the two groups. *P* values< 0.05 were considered statistically significant.

## Results

**Figure 3:**
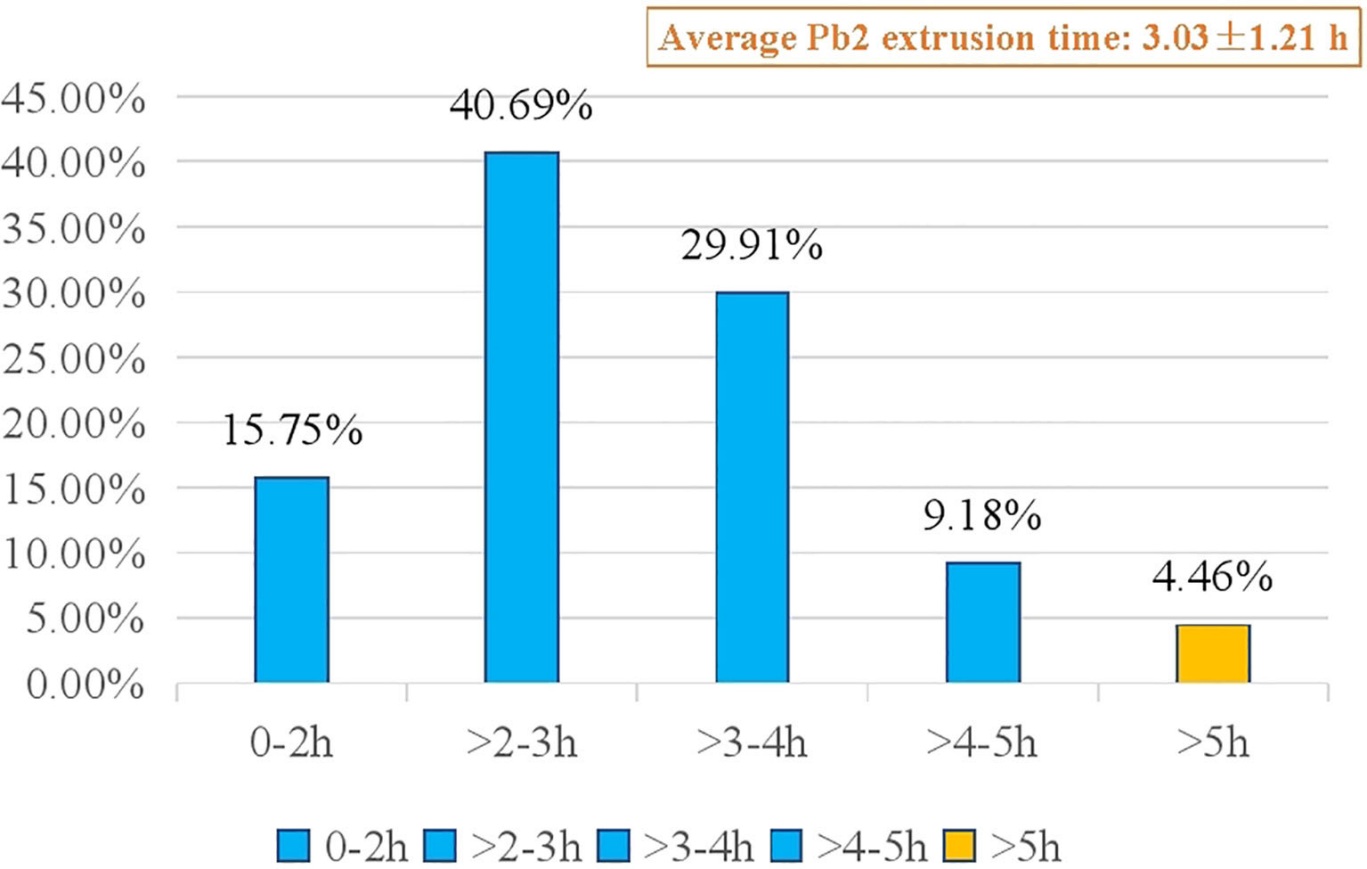
Proportion of different Pb2 extrusion time post-ICSI.

A total of 2480 MII oocytes from 278 ICSI cycles were screened for the analysis of fertilization prediction efficiency. In total, 74 degenerated oocytes post ICSI were excluded, and 2406 MII oocytes were ultimately monitored by time lapse. In total, 1949 (81.01%) MII oocytes had Pb2 extrusion, while 457 (18.99%) did not. The average time to Pb2 extrusion post ICSI was 3.03 ± 1.21 h, and the proportions of oocytes according to Pb2 extrusion times were as follows: 0-2 h (15.75%), >2-3 h (40.69%), >3-4 h (29.91%), >4-5 h (9.18%), and >5 h (4.46%) (**Figure 3**). The sensitivity, specificity, positive predictive value, and negative predictive value for fertilization prediction by Pb2 extrusion were 99.59%, 99.78%, 99.95% and 98.25%, respectively (**Table 1**). Pb2 extrusion time was correlated with blastocyst formation outcomes. The day 6 blastocyst formation and high-quality blastocyst formation rates in the >5 h group were only 7.14% and 3.57%, respectively, and were significantly lower than those in the 0-3 h and >3-5 h groups (**Table 2**).

**Table 1:** Efficiency analysis of fertilization prediction by Pb2 extrusion post-ICSI monitored by time lapse.

Index	Pb2 extrusion	Without Pb2 extrusion
No. of fertilized oocytes (n)	1948	8*
No. Of unfertilized oocytes (n)	1	449
Sensitivity (%)	99.59 (1948/1956)	
Specificity (%)	99.78 (449/450)	
Positive predictive value (%)	99.95 (1948/1949)	
Negative predictive value (%)	98.25 (449/457)	

*Three 2PN and five 1PN oocytes.

**Table 2:** The relationship between Pb2 extrusion time monitored by time lapse and fertilization or embryo development outcomes.

Group	Pb2 extrusion time			*P* value
	0~3 h	>3~5 h	>5 h	
Fertilization rate (%)	99.91 (1099/1100)	100.00 (762/762)	100.00 (87/87)	0.680
2PN fertilization rate (%)	96.45 (1061/1100)^a^	98.43 (750/762)	95.40 (83/87)	0.024
Day 3 high-quality embryo rate (%)	47.73 (505/1058)	44.91 (335/746)	43.37 (36/83)	0.421
Day 6 blastocyst formation rate (%)	65.97 (471/714)^bc^	54.01 (276/511)^d^	7.14 (4/56)	<0.001
Day 6 high-quality blastocyst formation rate (%)	40.34 (288/714)^ef^	28.18 (144/511)^g^	3.57 (2/56)	<0.001

^a^ represents P=0.013, compared with>3~5h group; ^b^ and ^c^ represents P<0.001 compared with >3~5h group and >5h group respectively, ^d^ represents P<0.001 compared with >5h group; ^e^ and ^f^ represents P<0.001 compared with >3~5h group and >5h group respectively, ^g^ represents P<0.001 compared with >5h group.

Fourteen unexpected fertilization failures and 20 unexpected low fertilization ICSI cycles were enrolled for EROA, and the patient characteristics were shown in **Table 3**. Baseline data were similar between the two groups, except for female age, which was significantly higher in the UFF group than in the ULF group (34.36 ± 4.92 vs. 31.30 ± 3.77, *P*=0.049), male factors were the major indication for ICSI in both groups(64.29 and 65.00%).

**Table 3:** Characteristics of the patients at baseline in 14 UFF and 20 ULF cycles.

Group	UFF cycles	ULF cycles	*P* value
No. of cycles (n)	14	20	/
Female age (years)	34.36 ± 4.92	31.30 ± 3.77	0.049
Male age (years)	35.93 ± 4.65	33.90 ± 5.65	0.277
Infertility duration (years)	3.71 ± 2.20	3.80 ± 2.42	0.904
BMI (kg/m2)	21.70 ± 3.00	21.06 ± 2.18	0.769
AMH (ng/ml)	2.25 ± 0.77	4.01 ± 4.56	0.158
AFC (n)	11.00 ± 3.90	14.65 ± 7.86	0.083
Indication for ICSI			
IVF fertilization failure history,n(%)	1/14 (7.14)	0/14 (0.00)	1.000
Combining male and female factors,n (%)	4/14 (28.57)	7/20 (35.00)	1.000
Male factor,n (%)	9/14 (64.29)	13/20 (65.00)	1.000
Obstructive Azoospermia,n (%)	6/14 (42.86)	4/20 (20.00)	/
Oligoasthenoteratozoospermia,n (%)	2/14 (14.29)	4/20 (20.00)	/
Oligoasthenozoospermia,n (%)	1/14 (7.14)	4/20 (20.00)	/
Necrozoospermia,n (%)	0/14 (0.00)	1/20 (5.00)*	/

BMI ,body mass index; AMH, anti-Mullerian hormone; AFC, antral follicle count; * Injected with spermatozoa obtained by testicular aspiration. “/” means without statistical analysis.

As to developmental and clinical outcomes of the 34 patients were presented in **Table 4**. Pb2 extrusion rate at 5h post ICSI was significantly lower in the UFF group than in the ULF group (0.00% vs. 32.21%, *P*<0.001). The fertilization rate and 2PN fertilization rate were both lower in the UFF group than in the ULF group, but the difference was not significant (71.74% vs. 77.88%, *P*=0.305; 58.70% vs. 64.42%, *P*=0.366). The proportions of fertilized or 2PN oocytes derived from EROA were both significantly higher in the UFF group than in the ULF group (100% vs. 61.73%, *P*<0.001; 100% vs. 58.21%, *P*<0.001). There were no significant differences in embryo development or clinical outcome parameters between the two groups. In general, in the UFF group, the day 3 high-quality embryo rate, day 6 blastocyst formation rate, and oocyte utilization rate were 42.59%, 48.28% and 34.78%, respectively, and in the ULF group, the rates were 51.15%, 49.45% and 36.06%, respectively. In the UFF group, 10 embryo transfer cycles were achieved by EROA, with a clinical pregnancy rate of 40.00% and implantation rate of 26.32%, while in the ULF group, 14 embryo transfer cycles with a clinical pregnancy rate of 57.14% and implantation rate of 37.04% were achieved.

**Table 4:** Developmental and clinical outcomes in 14 UFF and 20 ULF cycles after EROA treatment.

Group	UFF cycles	ULF cycles	*P* value
No. Of oocytes (n)	113	258	/
MII oocyte rate (%)	81.42(92/113)	80.62(208/258)	0.887
Oocyte degradation rate (%)	3.54(4/113)	1.55(4/258)	0.253
Pb2 extrusion rate at 5h(%)	0.00(0/92)	32.21(67/208)	<0.001
Fertilization rate (%)	71.74(66/92)	77.88(162/208)	0.305
Proportion of fertilized oocytes derived from EROA (%)	100(66/66)	61.73(100/162)	<0.001
2PN fertilization rate (%)	58.70(54/92)	64.42(134/208)	0.366
Proportion of 2PN fertilized oocytes derived from EROA (%)	100(54/54)	58.21(78/134)	<0.001
Cleavage rate (%)	95.45(63/66)	95.06(154/162)	1.000
Day 3 high-quality embryo rate (%)	42.59(23/54)	51.15(67/131)	0.333
Day 6 blastocyst formation rate (%)	48.28(14/29)	49.45(45/91)	1.000
Day 6 high-quality blastocyst formation rate (%)	34.48(10/29)	36.26(33/91)	1.000
Oocytes utilization rate (%)	34.78(32/92)	36.06(75/208)	0.896
No. Of Embryo transfer cycles(n)	10	14	
Clinical pregnancy rate (%)	40.00(4/10)	57.14(8/14)	0.680
Implantation rate (%)	26.32(5/19)	37.04(10/27)	0.533
Early miscarriage rate (%)	0.00(0/10)	7.14(1/14)	1.000
Ongoing pregnancy rate (%)	20.00(2/10)	15.38(2/13)	1.000
Live birth rate (%)	20.00(2/10)	35.71(5/14)	0.653

“/” means without statistical analysis.

The fertilization and embryological parameters derived from Pb2 extrusion or EROA oocytes were compared among the 34 cycles. As shown in **Table 5**, all the MII oocytes with Pb2 extrusion were derived from ULF cycles, and the fertilization rate, 2PN fertilization rate and oocyte utilization rate of oocytes derived from EROA in UFF and ULF cycles, respectively, were significantly lower than those of Pb2 extrusion oocytes (75.00 vs. 92.54%, *P*=0.005, 61.36 vs. 83.58%, *P*=0.003, 36.36 vs. 50.22%, *P*=0.023; 72.99 vs. 92.54%, *P*=0.001, 56.93 vs. 83.58%, *P*<0.001, 27.74 vs. 50.22%, *P*<0.001); however, there were no significant differences in day 3 embryo quality or blastocyst formation parameters between the groups.

**Table 5:** Comparison of fertilization and embryo development outcomes between Pb2 extrusion group and early rescue oocyte activation group in 14 UFF and 20 ULF cycles.

Group	Pb2 extrusion*	EROA in UFF cycles	EROA in ULF cycles	*P* value	
				EROA in UFF cycles vs. Pb2 extrusion	EROA in ULF cycles vs. Pb2 extrusion
No. of MII oocytes (n)	67	88	137	/	/
Fertilization rate (%)	92.54 (62/67)	75.00 (66/88)	72.99 (100/137)	0.005	0.001
2PN fertilization rate (%)	83.58 (56/67)	61.36 (54/88)	56.93 (78/137)	0.003	<0.001
1PN fertilization rate (%)	8.96 (6/67)	12.50 (11/88)	16.79 (23/137)	0.607	0.199
≥3PN fertilization rate (%)	1.49 (1/67)	1.14 (1/88)	0.00 (0/137)	1.00	0.328
Cleavage rate (%)	98.39 (61/62)	95.45 (63/66)	93.00 (93/100)	0.620	0.156
Day 3 high-quality embryo rate (%)	52.83 (28/53)	42.59 (23/54)	50.00 (39/78)	0.336	0.859
Day 6 blastocyst formation rate (%)	57.14 (16/28)	48.28 (14/29)	46.03 (29/63)	0.599	0.370
Day 6 high-quality blastocyst formation rate (%)	42.86 (12/28)	34.48 (10/29)	33.33 (21/63)	0.592	0.480
Oocytes utilization rate (%)	50.22 (37/67)	36.36 (32/88)	27.74 (38/137)	0.023	<0.001

*All the MII oocytes with Pb2 extrusion were derived from ULF cycles.

“/” means without statistical analysis.

There were also no significant differences in female age, male age or average number of transferred embryos between the Pb2 extrusion and EROA group. The biochemical pregnancy, clinical pregnancy and implantation rates were 70.00, 50.00, and 31.58%, respectively, in the Pb2 extrusion group and 57.14, 50.00, and 33.33%, respectively, in the EROA group, but there were no significant differences between the two groups (**Table 6**).

**Table 6:** Comparison of clinical outcomes between Pb2 extrusion group and early rescue oocyte activation group in 14 UFF and 20 ULF cycles.

Group	Pb2 extrusion	EROA	*P* value
Embryo transfer cycles (n)	10	14	/
Female age (year)	31.60 ± 3.50	32.64 ± 5.58	0.608
Male age (year)	34.10 ± 6.59	34.93 ± 5.20	0.734
Average no. of transferred embryo (n)	1.90 ± 0.32	1.93 ± 0.27	0.807
Biochemical pregnancy rate (%)	70.00 (7/10)	57.14 (8/14)	0.678
Clinical pregnancy rate (%)	50.00 (5/10)	50.00 (7/14)	1.000
Implantation rate (%)	31.58 (6/19)	33.33 (9/27)	1.000
Early miscarriage rate (%)	10.00 (1/10)	0.00 (0/14)	0.417
Ongoing pregnancy rate (%)	10.00 (1/10)	21.43 (3/14)	0.615
Live birth rate (%)	30.00 (3/10)	28.57 (4/14)	1.000

“/” means without statistical analysis.

## Discussion

The main purpose of this study was to establish a technique for the early prediction and early rescue of unfertilized oocytes to avoid unexpected fertilization failure or unexpected low fertilization in ICSI cycles. Our data showed that Pb2 extrusion monitored by time lapse was an accurate indicator in the prediction of fertilization failure, with a sensitivity and specificity of 99.59% and 99.78%, respectively. Five hours post ICSI was the appropriate time point for early rescue activation, which could achieve an acceptable fertilization rate, embryo development efficiency, and clinical outcomes.

The prediction of fertilization failure at an early stage is the foundation for EROA. At present, the observation of pronucleus formation at 16-18 hours after fertilization is the criterion for successful fertilization (11); however, rescue oocyte activation during this period has been proven to be inefficient due to the long *in vitro* culture time. Pb2 extrusion is a more valuable morphological indicator for the early prediction of fertilization and has been confirmed and applied in rescue ICSI of the IVF fertilization failure cycle (12, 13). However, a certain proportion of adjacent Pbs, fragmented Pbs or pseudo double Pbs made identification more complicated, especially in cases of partial fertilization failure (14), which could easily lead to misjudgment if the evaluated was based only on the shape of polar bodies at a single static time point. However, this limitation was overcome in this study by the use of time-lapse imaging to monitor the dynamic process of Pb2 extrusion from the cytoplasm (**Supplementary Video 2**). As shown in the results, the sensitivity and specificity of Pb2 extrusion to predict fertilization were 99.59% and 99.78%, respectively, while the positive predictive value and negative predictive value were 99.95% and 98.25%, respectively. It was worth noting that there are 8 fertilized oocytes (3 with 2PN and 5 with 1PN) in without Pb2 extrusion group (**Table 1**), this might be pathological events during the process of Pb2 extrusion, or the fragmented Pbs missed monitoring due to the 5min interval for time-lapse. Our results demonstrated that Pb2 extrusion monitored by time lapse can accurately identify unfertilized oocytes in the early stage of the fertilization process, which provides a basis for EROA.

Previous studies have demonstrated that rescuing oocyte activation at a late stage, such as day 1 or even later, has no clinical value due to poor embryonic outcomes (8, 15). Therefore, the earlier rescue oocyte activation performs, the more likely it is to achieve competitive clinical outcomes. To date, only one study (10) has attempted to perform rescue oocyte activation on day 0 after ICSI. The results indicated that the rescue activation oocyte implantation rate did not significantly differ from that of spontaneously activated oocytes. Our results also showed that EROA could reactivate unfertilized oocytes to achieve acceptable fertilization outcomes, while the embryo developmental parameters and clinical outcomes were not different from Pb2 extrusion oocytes, whether in UFF or ULF cycles. These data indicated that EROA is beneficial in maintaining the developmental and implantation potential of rescue-activated oocytes.

For the most appropriate time for EROA, in Shibahara’s study, the time for rescue activation was over 2.5-6 h from ICSI to ROA; however, based on our study, we recommend 5 h post ICSI because our retrospective data showed that the average time from ICSI to Pb2 extrusion was 3.03 ± 1.21 h, and only 56.44% of oocytes extruded Pb2 before 3 h. Therefore, some oocytes within “the process of fertilization” were at risk of unnecessary artificial activation if EROA was performed too early. Furthermore, in this study, 95.54% of oocytes showed Pb2 extrusion before 5 h, and the remaining 4.46% Pb2 after 5 h. The original developmental potential was poor (blastocyst formation rate of 7.14%, high-quality blastocyst formation rate of 3.57%), which might weaken the negative effect even with unnecessary artificial activation. Based on the above results, we suggest that the balance between early rescue activation and minimizing the risk of misjudgment is essential.

It should also be noted that the total oocyte utilization rate of EROA oocytes was significantly worse than that of spontaneously activated oocytes, which was due mainly to the relatively poor fertilization outcomes after EROA. All the EROA oocytes were derived from fertilization failure, which suggested a potential risk of functional abnormality. Studies have indicated that OAD could be due to a variety of factors, not only the deficiency of sperm factors but also abnormalities in crucial oocyte factors (2). In this study, we used ionomycin to activate oocytes by inducing a single calcium concentration rise; however, this treatment cannot solve all types of OAD, especially that caused by oocyte factors (16). Many studies conducted to date have attempted to optimize oocyte activation techniques, such as SrCl_2_ (17, 18), recombinant human PLCζ (19, 20) or a combination of two ionophores (21, 22). Overall activation methods that simulate physiologic calcium oscillations would be more efficient and would also be helpful towards improving the efficiency of EROA.

The most interesting aspect of this study was the reuse of fertilization failure oocytes during the current cycles, which could maximize the oocyte utilization rate in one single cycle. To our knowledge, the performance of oocyte activation in most cycles is based on the history of fertilization failure in previous cycles, which is limited by the lack of predictive indicators for fertilization failure, even for the mouse oocyte activation test (23) or PLCZ1 gene mutation analysis (24), which could indicate this in only a limited number of patients with fertilization failure caused by sperm-related factors. However, unexpected fertilization failures with unknown pathogeny still occurred from time to time, and if no treatments were attempted, precious oocytes would be wasted. In this study, most of the unexpected fertilization failures could be predicted and rescued by EROA in the UFF and ULF cycles, and the normal fertilization rate was 58.70% and 64.42%, with EROA contributing 100% and 58.21% in the normal fertilization, respectively. Precious oocytes could be fully utilized, which in turn reduced the medical burden for infertile patients. Notably, although the safety of AOA has been demonstrated (25–27), we still have little knowledge about the complex mechanisms of OAD and the downstream effects of AOA on fertilization and embryonic development; for this reason, AOA needs to be performed very cautiously in clinical practice.

The present study has some potential limitations. On the one hand, the data of this study were based on one single reproductive center and one single culture system, and it has been demonstrated that different culture systems have a significant effect on dynamic developmental parameters (28). Therefore, we recommend determining an optimal time point based on real data from one’s own laboratory before performing EROA, which is very useful for standardized clinical applications. On the other hand, the pathophysiological mechanisms of fertilization disorders are complex, and genetic defects in sperm or oocytes from infertile couples are also important factors leading to fertilization failure (24, 29, 30), which may not be resolved in these patients just with oocyte activation treatment. The present study did not conduct genetic testing for infertile couples, which might lead to some limitations in the study conclusions. Furthermore, we should note that the ICSI indications in the cases included in this study were mainly male factors (**Table 3**), while AOA was most effective in treating fertilization failure caused by sperm factors, which might also be the reason why acceptable fertilization outcomes could be obtained in this study.

In conclusion, this study suggested an effective strategy for early judgement and early rescue activation of fertilization failure oocytes, which could achieve acceptable fertilization, embryo development and clinical outcomes to avoid unexpected fertilization failure or low fertilization cycles.

## Data availability statement

The original contributions presented in the study are included in the article/**Supplementary Material**. Further inquiries can be directed to the corresponding authors.

## Ethics statement

The studies involving humans were approved by ethics committee of the People’s Hospital of Guangxi Zhuang Autonomous Region(Approval No: LL-KY-SY-2021-16). The studies were conducted in accordance with the local legislation and institutional requirements. The participants provided their written informed consent to participate in this study.

## Author contributions

LX: Funding acquisition, Methodology, Project administration, Supervision, Validation, Visualization, Writing – original draft, Writing – review & editing. SW: Data curation, Formal Analysis, Writing – original draft. PW: Data curation, Methodology, Writing – original draft. HL: Formal Analysis, Methodology, Writing – original draft. XM: Investigation, Software, Supervision, Writing – original draft. JQ: Data curation, Project administration, Writing – original draft. YL: Software, Validation, Writing – original draft. XZ: Methodology, Writing – original draft. ZL: Methodology, Resources, Writing – original draft. YH: Resources, Software, Writing – original draft. LC: Resources, Software, Writing – original draft. WS: Resources, Writing – original draft. LL: Project administration, Resources, Writing – original draft.

## References

[1] Ebner T Montag M Montag M van der Ven K van der Ven H Ebner T. Live birth after artificial oocyte activation using a ready-to-use ionophore: a prospective multicentre study. Reprod Biomedicine Online (2015) 30(4):359–65. doi: 10.1016/j.rbmo.2014.11.012 25596904

[2] Yeste M Jones C Amdani SN Patel S Coward K. Oocyte activation deficiency: a role for an oocyte contribution? Hum Reprod Update (2016) 22(1):23–47. doi: 10.1093/humupd/dmv040 26346057

[3] ESHRE Special Interest Group of Embryology and Alpha Scientists in Reproductive Medicine. The Vienna consensus: report of an expert meeting on the development of ART laboratory performance indicators. Reprod Biomedicine Online (2017) 35(5):494–510. doi: 10.1016/j.rbmo.2017.06.015 28784335

[4] Kashir J Ganesh D Jones C Coward K. Oocyte activation deficiency and assisted oocyte activation: mechanisms, obstacles and prospects for clinical application. Hum Reprod Open (2022) 2022(2):hoac003. doi: 10.1093/hropen/hoac003 35261925 PMC8894871

[5] Campos G Sciorio R Esteves SC. Total fertilization failure after ICSI: insights into pathophysiology, diagnosis, and management through artificial oocyte activation. Hum Reprod Update (2023) 29(4):369–94. doi: 10.1093/humupd/dmad007 36977357

[6] Akashi K Yamada M Jwa SC Utsuno H Kamijo S Hirota Y. Artificial oocyte activation using Ca2+ ionophores following intracytoplasmic sperm injection for low fertilization rate. Front In Endocrinol (2023) 14:1131808. doi: 10.3389/fendo.2023.1131808 PMC10034378 36967799

[7] Sfontouris IA Nastri CO Lima MLS Tahmasbpourmarzouni E Raine-Fenning N Martins WP. Artificial oocyte activation to improve reproductive outcomes in women with previous fertilization failure: a systematic review and meta-analysis of RCTs. Hum Reprod (Oxford England) (2015) 30(8):1831–41. doi: 10.1093/humrep/dev136 26082476

[8] Lu Q Zhao Y Gao X Li Y Ma S Mullen S. Combination of calcium ionophore A23187 with puromycin salvages human unfertilized oocytes after ICSI. Eur J Obstetrics Gynecology Reprod Biol (2006) 126(1):72–6. doi: 10.1016/j.ejogrb.2005.10.038 16352389

[9] Sakurai M Watanabe S Tanaka T Matsunaga R Yamanaka N Kamihata M. Effect of artificial oocyte activation by calcium ionophore on one-day-old unfertilized oocytes after ICSI. J Mamm Ova Res (2015) 32(3):115–20. doi: 10.1274/jmor.32.115

[10] Shibahara T Fukasaku Y Hayashi N Miyazaki N Kawato H Minoura H. Early rescue oocyte activation for activation-impaired oocytes with no second polar body extrusion after intracytoplasmic sperm injection. J Assisted Reprod Genet (2021) 38(5):1061–8. doi: 10.1007/s10815-021-02089-1 PMC8190240 33619678

[11] Alpha Scientists in Reproductive Medicine and ESHRE Special Interest Group of Embryology. The Istanbul consensus workshop on embryo assessment: proceedings of an expert meeting. Hum Reproduction(Oxford England) (2011) 26(6):1270–83. doi: 10.1093/humrep/der037 21502182

[12] Chen C Kattera S. Rescue ICSI of oocytes that failed to extrude the second polar body 6 h post-insemination in conventional IVF. Hum Reprod (Oxford England) (2003) 18(10):2118–21. doi: 10.1093/humrep/deg325 14507831

[13] Huang B Qian K Li Z Yue J Yang W Zhu G. Neonatal outcomes after early rescue intracytoplasmic sperm injection: an analysis of a 5-year period. Fertility Sterility (2015) 103(6):1432–1437.e1431. doi: 10.1016/j.fertnstert.2015.02.026 25813286

[14] Guo Y Liu W Wang Y Pan J Liang S Ruan J. Polarization microscopy imaging for the identification of unfertilized oocytes after short-term insemination. Fertility Sterility (2017) 108(1):78–83. doi: 10.1016/j.fertnstert.2017.05.009 28600104

[15] Esbert M Carmody A Ballesteros A Seli E Scott RT. Calcium ionophore a23187 treatment to rescue unfertilized oocytes: a prospective randomized analysis of sibling oocytes. Reprod Biomedicine Online (2022) 45(5):878–83. doi: 10.1016/j.rbmo.2022.06.021 36038485

[16] Vanden Meerschaut F Nikiforaki D De Gheselle S Dullaerts V Van den Abbeel E Gerris J. Assisted oocyte activation is not beneficial for all patients with a suspected oocyte-related activation deficiency. Hum Reprod (Oxford England) (2012) 27(7):1977–84. doi: 10.1093/humrep/des097 22493027

[17] Kim J-W Kim S-D Yang S-H Yoon S-H Jung J-H Lim J-H. Successful pregnancy after SrCl2 oocyte activation in couples with repeated low fertilization rates following calcium ionophore treatment. Syst Biol In Reprod Med (2014) 60(3):177–82. doi: 10.3109/19396368.2014.900832 24645921

[18] Fawzy M Emad M Mahran A Sabry M Fetih AN Abdelghafar H. Artificial oocyte activation with SrCl2 or calcimycin after ICSI improves clinical and embryological outcomes compared with ICSI alone: results of a randomized clinical trial. Hum Reprod (Oxford England) (2018) 33(9):1636–44. doi: 10.1093/humrep/dey258 30099496

[19] Yoon S-Y Eum JH Lee JE Lee HC Kim YS Han JE. Recombinant human phospholipase C zeta 1 induces intracellular calcium oscillations and oocyte activation in mouse and human oocytes. Hum Reproduction(Oxford England) (2012) 27(6):1768–80. doi: 10.1093/humrep/des092 22456923

[20] Sanusi R Yu Y Nomikos M Lai FA Swann K. Rescue of failed oocyte activation after ICSI in a mouse model of male factor infertility by recombinant phospholipase Cζ. Mol Hum Reprod (2015) 21(10):783–91. doi: 10.1093/molehr/gav042 PMC4586348 26187950

[21] Nakagawa K Yamano S Moride N Yamashita M Yoshizawa M Aono T. Effect of activation with Ca ionophore A23187 and puromycin on the development of human oocytes that failed to fertilize after intracytoplasmic sperm injection. Fertility Sterility (2001) 76(1):148–52. doi: 10.1016/s0015-0282(01)01839-8 11438334

[22] Gao Y Yang D Fang Y Wang X Li D. Live birth following an innovative artificial oocyte activation protocol using double calciumstimulators. Chin Med J (2023) 136(17):2101–3. doi: 10.1097/CM9.0000000000002407 PMC10476707 36914951

[23] Vanden Meerschaut F Leybaert L Nikiforaki D Qian C Heindryckx B De Sutter P. Diagnostic and prognostic value of calcium oscillatory pattern analysis for patients with ICSI fertilization failure. Hum Reprod (Oxford England) (2013) 28(1):87–98. doi: 10.1093/humrep/des368 23081875

[24] Sang Q Ray PF Wang L. Understanding the genetics of human infertility. Science (2023) 380(6641):158–63. doi: 10.1126/science.adf7760 37053320

[25] Capalbo A Ottolini CS Griffin DK Ubaldi FM Handyside AH Rienzi L. Artificial oocyte activation with calcium ionophore does not cause a widespread increase in chromosome segregation errors in the second meiotic division of the oocyte. Fertility Sterility (2016) 105(3):807–814.e2. doi: 10.1016/j.fertnstert.2015.11.017 26658129

[26] Shebl O Trautner PS Enengl S Reiter E Allerstorfer C Rechberger T. Ionophore application for artificial oocyte activation and its potential effect on morphokinetics: a sibling oocyte study. J Assisted Reprod Genet (2021) 38(12):3125–33. doi: 10.1007/s10815-021-02338-3 PMC8666403 34642877

[27] Zhang X Li L Zhang W Luo Y Mao Y Du H. Embryo development and live birth resulted from artificial oocyte activation after microdissection testicular sperm extraction with ICSI in patients with non-obstructive azoospermia. Front In Endocrinol (2023) 14:1123541. doi: 10.3389/fendo.2023.1123541 PMC9989460 36896176

[28] Liu Y Qi F Matson P Morbeck DE Mol BW Zhao S. Between-laboratory reproducibility of time-lapse embryo selection using qualitative and quantitative parameters: a systematic review and meta-analysis. J Assisted Reprod Genet (2020) 37(6):1295–302. doi: 10.1007/s10815-020-01789-4 PMC7311559 32361919

[29] Xu M Wu W Zhao M Chung J Li TC Chan D. Common dysmorphic oocytes and embryos in assisted reproductive technology laboratory in association with gene alternations. Int J Biochem Cell Biol (2022) 152:106298. doi: 10.1016/j.biocel.2022.106298 36122887

[30] Wei Y Wang J Qu R Zhang W Tan Y Sha Y. Genetic mechanisms of fertilization failure and early embryonic arrest: a comprehensive review. Hum Reprod Update. (2023). doi: 10.1093/humupd/dmad026 37758324

